# Supplementary material for: Cells on Hydrogels with Micron-Scaled Stiffness Patterns Demonstrate Local Stiffness Sensing
Source: Nanomaterials (Basel). 2022 Feb 15;12(4):648. doi: 10.3390/nano12040648 (PMC8880377; doi:10.3390/nano12040648)
Supplement: Supplementary file 1 [file nanomaterials-12-00648-s001.zip › SI_mgharbel.pdf]

Supporting Information

Cells on hydrogels with micron-scaled stiffness patterns demonstrate local stiffness sensing

Abbas Mgharbel[1,2†], Camille Migdal[1,2,3†], Nicolas Bouchonville[1], Paul Dupenloup[1], David Fuard[1], Eline Lopez-Soler[1,3], Marie Courçon[1,2], Caterina Tomba[1,5], Danielle Gulino-Debrac[1,2], Hélène Delanoë-Ayari[4], and Alice Nicolas [*1]

[1]Univ. Grenoble Alps, CNRS, LTM, 38000 Grenoble, France
[2]Univ. Grenoble Alps, CEA, CNRS, Inserm, BIG-BCI, 38000 Grenoble, France
[3]Univ. Grenoble Alps, CEA, Inserm, BIG-BGE, 38000 Grenoble, France
[4]Univ. Claude Bernard Lyon1, CNRS, Institut Lumière Matière, 69622 Villeurbanne, France
[5]Univ. Grenoble Alps, CNRS, Grenoble INP, Institut Néel, 38000 Grenoble, France

† These authors contributed equally to this work.

[*]Corresponding author: alice.nicolas@cea.fr

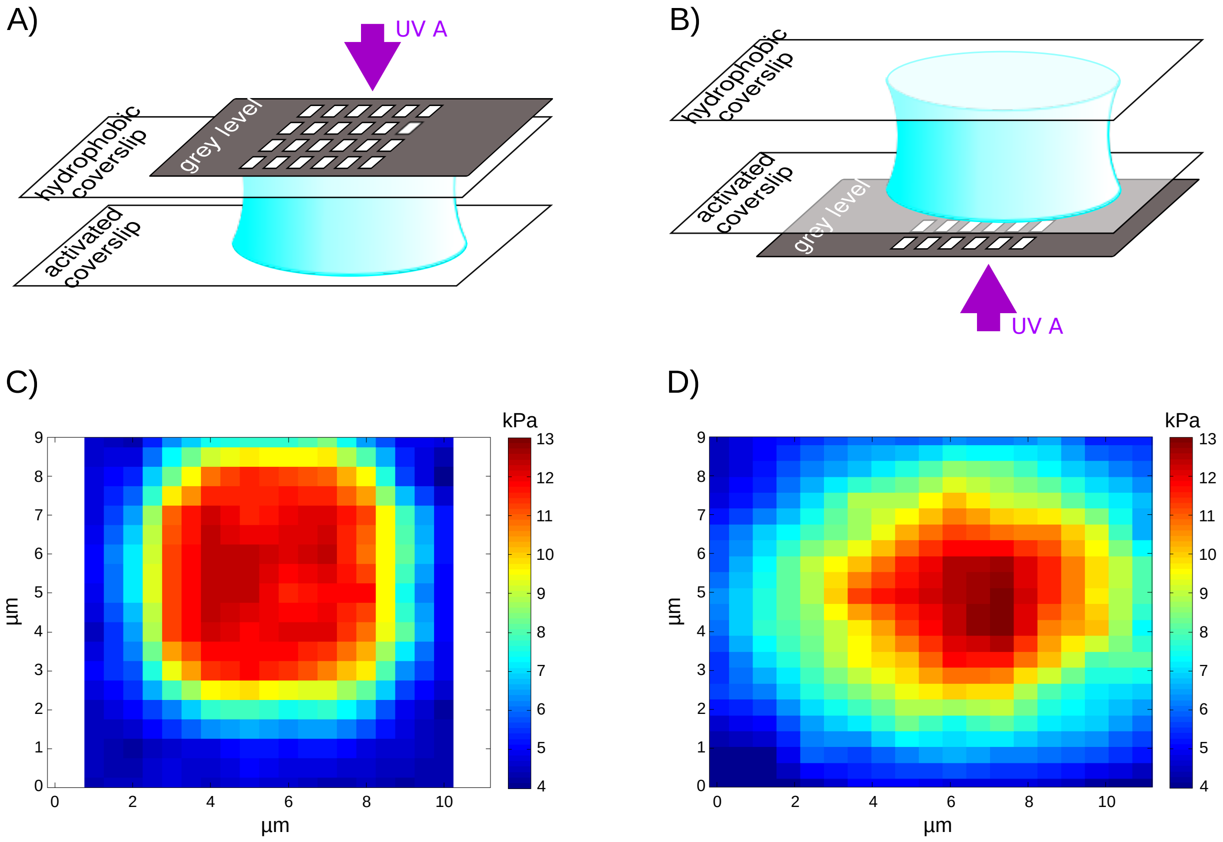

Figure S1: Comparison of the Young's modulus of the hydrogel on the top and the bottom surfaces. (A) The grey level mask is in contact with the top surface. (B) The light comes first to the bottom surface through the grey level mask. A non adherent coverslip that will be removed after polymerization guarantees that the top surface is flat. (C,D) Young's moduli for dots with a diameter of 5 µm and a spacing of 10 µm obtained with setups (A) or (B) respectively.

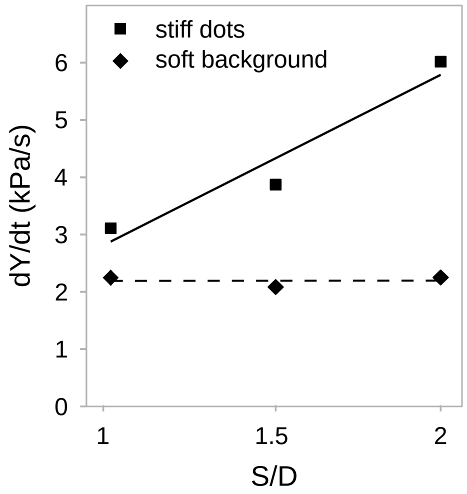

Figure S2: Influence of the geometry of the pattern on the sensitivity of the local stiffness of the hydrogel to the UV exposure time. D is the width of the transparent dots on the photomask and S their spacing. D = 5 µm.

A)

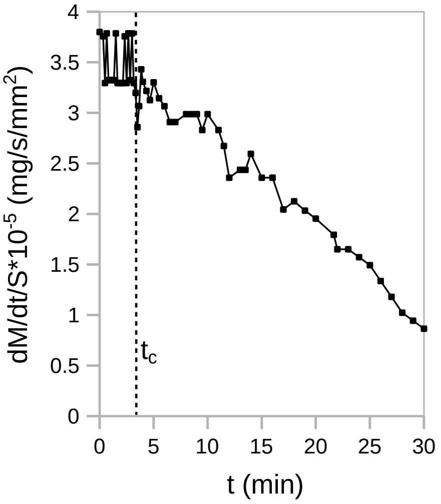

B)

Y (kPa)	tc (min)
3.3	3.3+/-0.3
11.8	6.5+/-0.3
24.7	8.1+/-0.2

Figure S3: Stiffness dependence of the kinetics of the dehydration of a polyacrylamide hydrogel. (A) Kinetics of the loss of mass of a gel of rigidity 3.3 kPa at 21°C. When $t < t_c$, the water evaporates from the surface at a constant rate. For $t > t_c$, the water evaporates with a decreasing rate, by diffusing in the volume of the gel through the network of pores [81]. (B) Stiffness dependence of t_c.

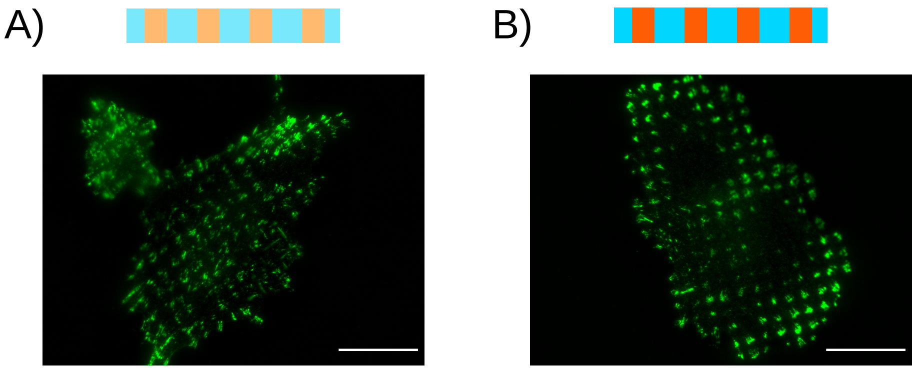

Figure S4: Paxillin proteins condense on the stiff dots also when the surface density of the fibronectin is 1.7 µg/cm^2. The dots are 3 µm wide and spaced by 3 µm. (A) Stiff dots: 1.9 ± 0.2 kPa, soft background 0.4 ± 0.1 kPa. (B) Stiff dots: 5.6 ± 0.4 kPa, soft background 1.7 ± 0.2 kPa. Bars 30 µm.

Table S1: Geometry and stiffness conditions of the patterned hydrogels exploited for TFM analysis.

Dot diameter (µm)	Spacing (µm)	Y_{stiff} (kPa)	Y_{soft} (kPa)
3.5	4	0.7±0.1	0.4±0.1
4	5	5.9±0.4	3.7±0.9
5	7.5	8.4±0.4	4.0±0.6
3.5	4	9.1±1.2	5.1±0.9
2	4	12.4±1.0	6.7±2.1

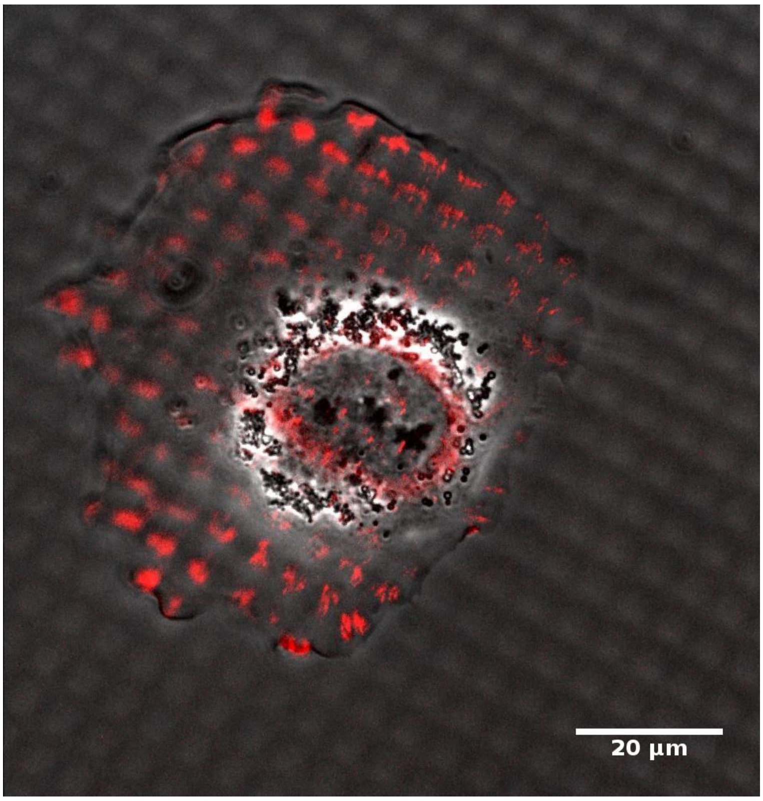

Video S1: Paxillin-stained REF-52 cell plated on a pattern of rigidity with dots of 3 µm spaced by 3 µm and rigidities of 14.3±0.8 kPa and 7.2±1 kPa. Fibronectin coating 0.6 µg/cm^2. Timelapse 10 min.
