# Supplementary figures and images for: Cells on Hydrogels with Micron-Scaled Stiffness Patterns Demonstrate Local Stiffness Sensing
Source: Nanomaterials (Basel). 2022 Feb 15;12(4):648. doi: 10.3390/nano12040648 (PMC8880377; doi:10.3390/nano12040648)

A)

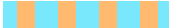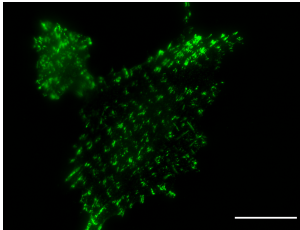

B)

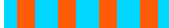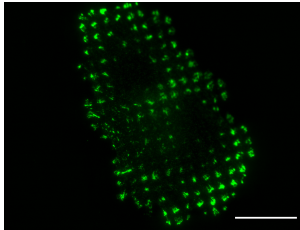

Supplement: Supplementary file 1 [file nanomaterials-12-00648-s001.zip › FigSI_1_7ugcm2.pdf]

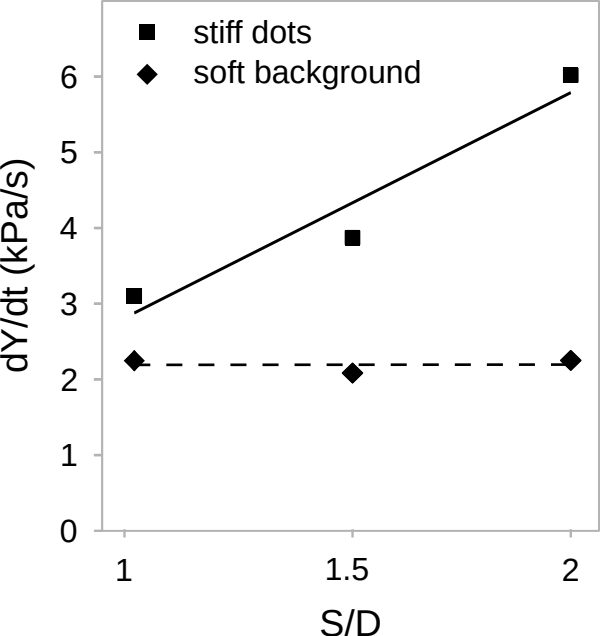

Supplement: Supplementary file 1 [file nanomaterials-12-00648-s001.zip › FigSI_dYdt.pdf]

A)

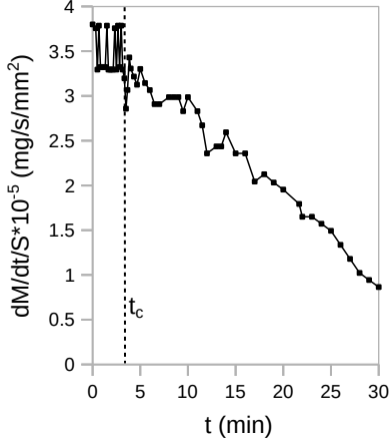

B)

| Y (kPa) | t <sub>c</sub> (min) |
|---------|----------------------|
| 3.3     | 3.3+/-0.3            |
| 11.8    | 6.5+/-0.3            |
| 24.7    | 8.1+/-0.2            |

Supplement: Supplementary file 1 [file nanomaterials-12-00648-s001.zip › FigSI_dehydrat.pdf]

A)

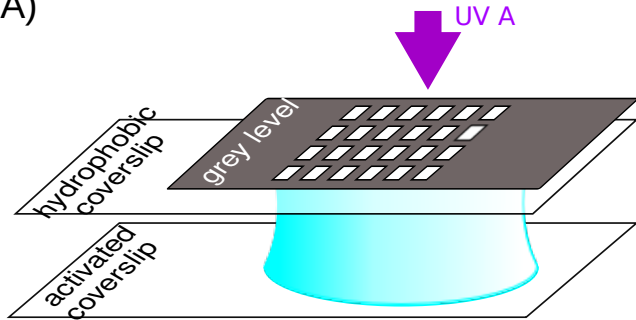

B)

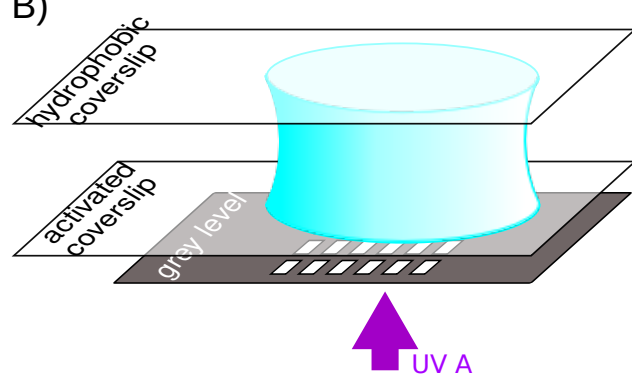

C)

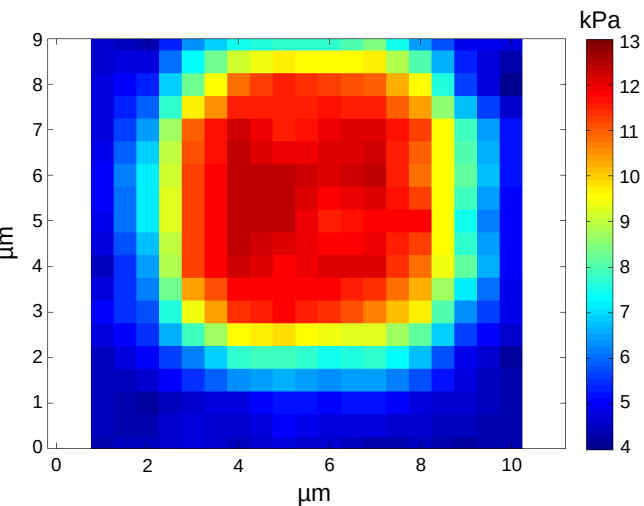

D)

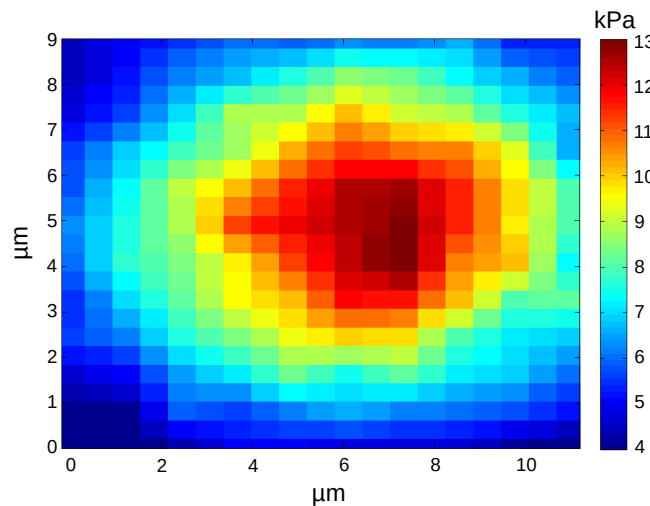

Supplement: Supplementary file 1 [file nanomaterials-12-00648-s001.zip › FigSI_inverse_setup.pdf]

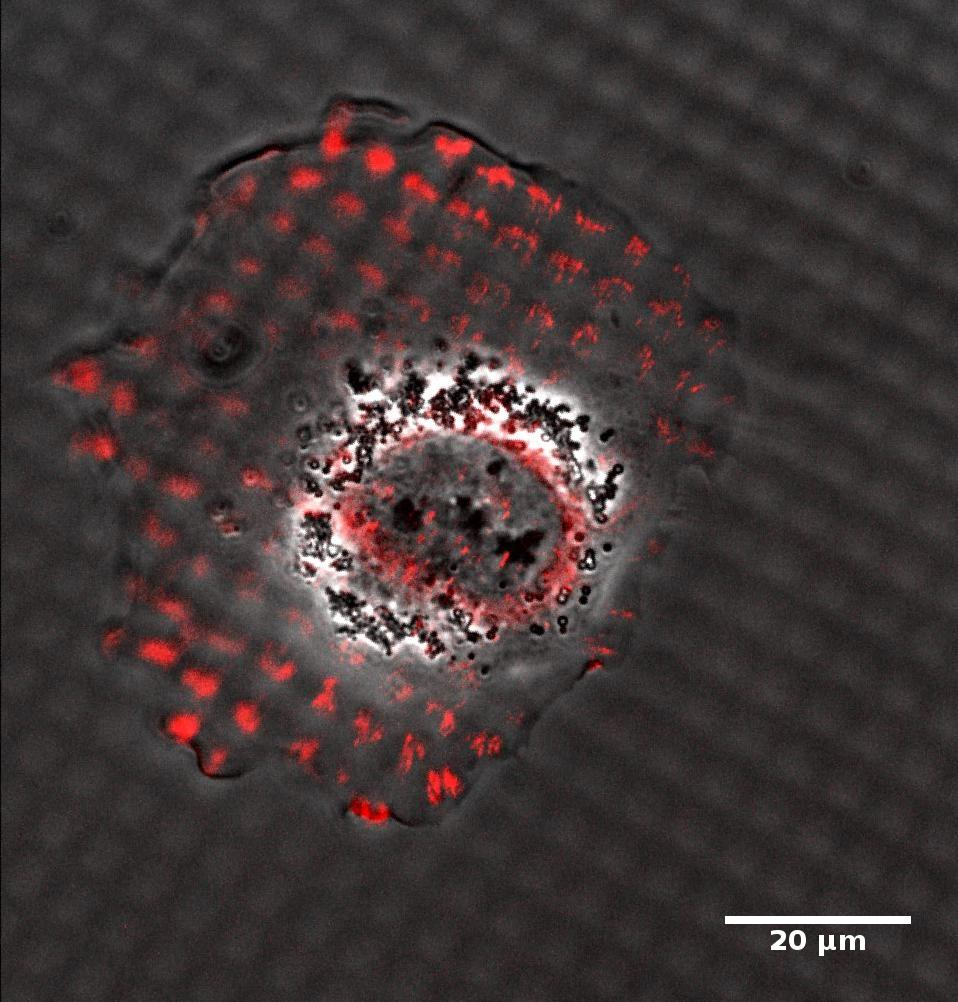

Supplement: Supplementary file 1 [file nanomaterials-12-00648-s001.zip › SImovie.png]
